# The Politics of LGBT+ Health Inequality: Conclusions from a UK Scoping Review

**DOI:** 10.3390/ijerph18020826

**Published:** 2021-01-19

**Authors:** Elizabeth McDermott, Rosie Nelson, Harri Weeks

**Affiliations:** 1 Department of Health Research, Lancaster University, Lancashire LA1 4YW, UK; r.b.nelson@lancaster.ac.uk; 2 The National LGB&T Partnership, Exeter EX4 6NA, UK; harri.weeks@gmail.com

**Keywords:** LGBT, health, inequality, scoping review, UK, mental health, palliative care, end of life care, cancer

## Abstract

This scoping review of UK evidence aimed to describe what is known about Lesbian, Gay, Bisexual, and Trans (LGBT+) health inequalities in relation to cancer, mental health, and palliative care to inform research, policy and public health interventions. Using a scoping review methodology, we identified studies from database searches, citation tracking, and expert consultation. The in/exclusion criteria was based on the PICOS framework. The data were charted and then summarised to map the theoretical approaches and the main types of evidence and identify knowledge gaps. In total, 279 articles were screened and 83 were included in the final review. We found that there is limited UK research examining LGBT+ health inequality in cancer, mental health and palliative care. We would argue that this thin evidence base is partly due to national policy discussions of LGBT+ health inequality that are framed within a depoliticised ‘it’s getting better’ narrative, and an unwillingness to adequately acknowledge the unjust social and economic relations that produce LGBT+ health inequality. In addition, LGBT+ health inequality is depoliticised by existing public health explanatory theories, models and frameworks that exclude sexual orientation and gender diversity as dimensions of power that interlock with those of socio-economic, race and ethnicity. This is a barrier to developing public health interventions that can successfully tackle LGBT+ health inequality

## 1. Introduction

There is now a body of research evidence that demonstrates that lesbian, gay, bisexual, and trans (LGBT+) people experience significant health inequalities in terms of health outcomes, health care service provision and health risk factors in comparison to cis-heterosexual populations [1]. Recent research has highlighted how LGBT+ experiences of health and well-being require specific or specialised identity-centred interventions to better support LGBT+ health in a range of specialties [1,2,3,4,5,6]. Current research across multiple types of service provision has highlighted how LGBT+ people perceive current health or social care provision as in need of improvement in relation to their treatment and sexual and/or gender identity [7,8,9].

Increasingly, these inequities have been recognised by the UK Government and national health bodies (e.g., NHS England and Public Health England). In particular, policy attention on LGBT+ health was sparked by the 2017 Government Equalities Office survey of UK-based LGBT+ people [1]. This survey received more than 108,000 responses from LGBT+ people who highlighted the significant everyday experiences of discrimination that LGBT+ people live through [1]. One outcome from the survey was the UK Government 2018 *LGBT Action Plan* with a specific target of improving health policy and health care provision for LGBT+ people [1]. Since then, the UK Government Women and Equalities Committee launched an inquiry into LGBT+ health and social care and made policy recommendations based on their findings [10]. However, preliminary work to define this scoping review showed that very few actionable suggestions have been made, with most policy and health care strategies calling for more research into LGBT+ health inequalities. 

Critics have suggested that the reluctance to develop policies to seriously tackle LGBT+ inequality is explained less by the absence of robust evidence and instead more likely to demonstrate the entrenched resistance to LGBT+ equality in the UK [11]. We would argue that policy discussions of LGBT+ health inequality are ‘depoliticised’ because they are framed by a ‘it’s getting better’ narrative [12] and an unwillingness to adequately acknowledge the unjust social and economic relations that produce LGBT+ health inequality. In addition, LGBT+ health inequality is depoliticised by existing public health explanatory theories, models and frameworks that exclude sexual orientation and gender diversity as dimensions of power that interlock with those of socio-economic, race and ethnicity, etc. [13]. For example, neither the influential Social Determinants of Health ‘Rainbow Model’ [14] nor Ecosocial Theory [15] include sexual orientation or gender diversity in their explanations of health inequality. 

Against this background, the aim of this scoping review study was to draw together the LGBT Health Advisor at NHS England, policy makers, LGBT+ third-sector organisations and academics to consider the type of research required to develop policy and practice interventions to tackle LGBT+ health inequality. The aim of this study was to: (a) identify UK research on LGBT+ health inequalities to support policy development aimed at reducing LGBT+ health inequalities; (b) to develop recommendations on future health research and policy that aim to reduce LGBT+ health inequalities.

The focus of the review was developed in collaboration following a discussion of the strategic direction of key national health care policies, government equality policy and the UK research councils. The decision was taken to concentrate on cancer, mental health and palliative care because these were national health priorities where there was some recognition, in both policy and research, of the inequalities faced by LGBT+ populations. Collaborative meetings resulted in the following research question: What is known from the existing UK research literature and policy on LGBT+-relevant risk factors, prevention strategies, and health care experiences across (a) cancer, (b) mental health, and (c) palliative care? 

## 2. Methods

This study used a scoping review methodology which is a more recent addition to evidence synthesis methods [16] but has been gaining increased interest in health research [16,17,18]. Scoping reviews are conducted for different purposes than systematic reviews. The purpose of scoping reviews is to ‘describe the nature of a research field rather than synthesise findings’ [19]. Scoping reviews can be conducted to identify knowledge gaps, scope a body of literature, clarify concepts, set research agendas or provide a roadmap for a subsequent full systematic review [16,17,18].

Despite the different purpose to systematic reviews, scoping reviews follow a similar structured approach and use rigorous transparent methods [19]. Since Arksey and O’Malley [20] developed the framework for scoping reviews, there has been growing acknowledgement of the need to establish methodological standardisation and reporting guidelines [16,17,18]. This scoping review follows the five recommended steps in completing scoping reviews [17,18,20]: (a) identifying the research question; (b) identifying relevant studies; (c) study selection; (d) charting the data; (e) collating, summarising and reporting the results. 

### 2.1. Search Strategy

Studies were identified through electronic database searches, reference citation, online grey literature searches and expert consultation. The electronic database searches were restricted to PubMed and Web of Science databases to ensure both clinical and social science research were located. Experts in each subfield (cancer, mental health, and palliative care) were contacted with preliminary database findings to gain clarity on any papers that had not emerged during the database search. 

The search terms were adapted for each electronic database (see Table 1 and Table 2) but included 3 domains: sexual and gender identity; health condition; and geographical location. Searches were conducted across cancer, mental health, and palliative care using either keywords or ‘mesh’ searches depending on database. 

### 2.2. Study Selection

The PICOS framework was utilised to define the inclusion/exclusion criteria (see Table 3) which is recommended when time and resources are limited [21]. Two reviewers screened the titles and abstracts from the electronic databases and then applied the inclusion/exclusion criteria to the full-text papers. In the course of conducting the scoping review, two reviewers made additional decisions to exclude from the review medical case studies and treatment guidelines because they did not address population health concerns. The final included studies were collated in the Endnote reference management system. Two reviewers independently checked the inclusion and exclusion criteria against all studies found in the course of database searches, citation tracking, journal hand searching, and expert consultation.

### 2.3. Data charting and Summarising Results

All data were reported according to PRISMA guidelines. A scoping review does not synthesise results but rather present an overview of all material reviewed [20]. Data were charted to allow for a narrative review. Arksey and O’Malley [20] borrow the term ‘charting’ to refer to Ritchie and Spencer’s [22] technique for synthesising and interpreting qualitative data by filtering, categorising and organising material according to key issues and themes. 

In this scoping review, two reviewers designed a standardised data chart excel template that allowed for data to be both extracted from each full-text study, and to develop an overall narrative review. The data chart template contained the main study details (e.g., study population, aim, methodology, key results) but also included ‘conceptual framework’ and ‘interpretation’ categories. The characteristics for each full text article was charted by a single reviewer with a second reviewer regularly assessing the data charting process to resolve any conflicts. This process was used to develop an overall narrative that summarised the results in a way that was consistent with the scoping review research question and purpose [17].

## 3. Results

### 3.1. Search Results

**Figure 1:**
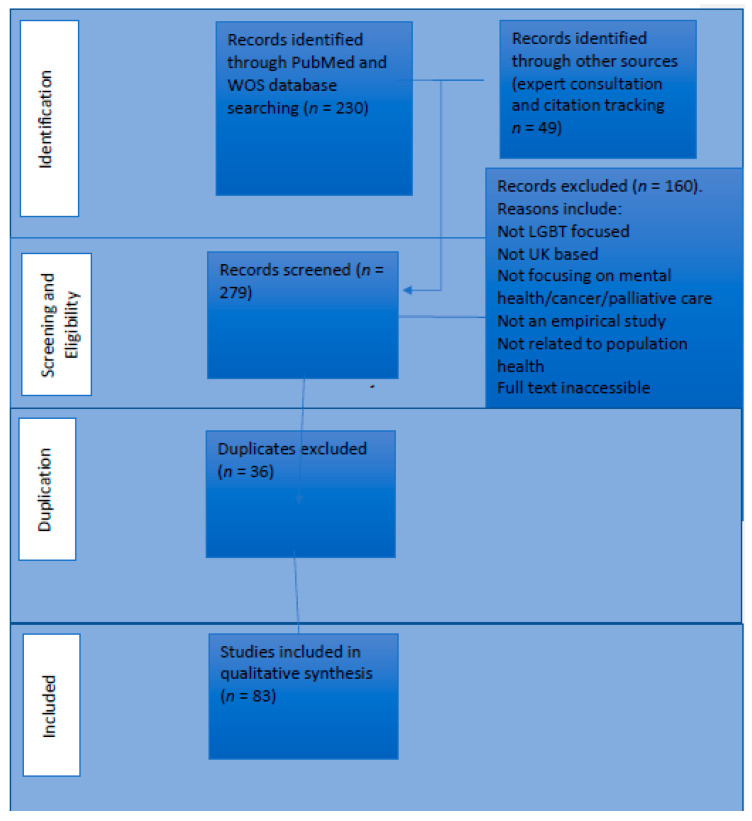
Search results.

The search was conducted between February and March 2020. In total, 279 articles were screened and 83 studies were included in the final review (see Figure 1 for PRISMA flowchart and Appendix A for included studies and a data extraction chart). 

### 3.2. Results Summary

In this section, we summarise the broad UK research trends in relation to understanding LGBT+ health inequalities in (a) cancer, (b) palliative care, and (c) mental health. 

### 3.3. Cancer

The scoping review yielded 31 studies relevant to LGBT+ cancer care in the United Kingdom. Of these studies, 12 focused on anal and/or prostate cancer [23,24,25,26,27,28,29,30,31,32,33,34], 2 focused on cervical cancer [35,36], 5 focused on unspecified cancers [37,38,39,40,41], 3 focused on breast cancer [42,43,44], and 9 focused on HPV-related work [45,46,47,48,49,50,51,52,53]. These studies used a range of methods including qualitative studies (7) [26,32,38,39,42,49,51], quantitative studies (9) [28,35,40,41,44,47,48,50,52], systematic reviews (2) [43,54], meta-syntheses (2) [23,31], mixed-methods studies (1) [45], summit papers (1) [37], cost-efficacy papers (2) [24,29], clinical papers (4) [25,33,46,55], and literature reviews (3) [30,36,53]. Overall, these studies are largely focused on sexual organs, i.e., HPV, AIDS, cervical cancer, anal cancer, and prostate cancer. 

Each paper represented different foci of interest. A total of 7 papers looked at LGBT+-specific screening, cost efficiencies, prevalence and risk factors [24,29,41,43,46,47,53]. A total of 8 papers looked at LGBT+ patient experiences pre, during, and/or post-treatment [23,26,31,32,35,38,39,40]. A total of 11 studies explored LGBT+ people’s cancer awareness and the efficacy/acceptability of treatments/testing/screening [25,27,28,33,36,42,44,45,50,51,54]. A total of 5 studies focused on health provider and expert attitudes towards LGBT+ patients’ treatment and diagnoses [30,37,48,49,52].

The results from all studies indicated that LGBT+ people were more likely to have a negative experience or outcome when being diagnosed, receiving treatment, or in post-treatment in comparison to the cis-heterosexual population. The majority of authors acknowledged that LGBT+ people’s poorer experience and outcomes were due to the absence of LGBT+-specific care and attention from health care providers. Qualitative studies highlighted a significant area of concern was that LGBT+ people struggle to ‘come out’ in a cancer treatment setting [39], and consequently not receiving culturally competent care [32,35,40,42,44,56]. Fish and Williamson’s work [38] found, for example, that some LGB people hid their sexual identities during cancer care treatment and in support environments [38]. Some studies indicated heteronormative institutional practices that implicitly marginalised LGBT+ people, such as Doran’s study, which demonstrated how gay men with prostate cancer were treated with heteronormative assumptions that did not meet their needs [26]. 

The absence of UK large-scale comparative data and epidemiological data was a deficiency in the research studies identified. The implications of this lack of data were underlined by expert consultants as a key problem in developing understandings of cancer prevalence and recovery rates in LGBT+ populations. It was suggested that including sexual identity and gender diversity characteristics in the cancer registry would enable the study of cancer prevalence and epidemiology in LGBT+ populations. Experts also raised issues regarding the underfunding of LGBT+ cancer scholarship and barriers, i.e., ‘institutional homophobia’ in attempting to access data, gain funding, and enact health care policies to better support LGBT+ cancer patients. 

### 3.4. Palliative Care

The scoping review provided 10 articles relevant to palliative care and LGBT+ people in the UK. A total of 8 papers were qualitative studies [8,57,58,59,60,61,62,63] and two papers were systematic reviews [7,64]. One paper explored how older LGB individuals felt about the ‘right to die’ [60], two systematic reviews assessed current work in bereavement, palliative care, and palliative treatments [7,64], and seven qualitative studies assessed the experience of loss and bereavement for gay and lesbian elders [8,57,58,59,61,62,63]. 

All 10 studies demonstrated that palliative care and bereavement considerations were different for LGBT+ older people. Almack et al.’s [57] study found, for example, that older LGBT+ people may have had to live closeted lives and therefore may not have their same-gender partners recognised after their deaths [57]. Ingham et al. [59] found that older LGB women may face complex barriers following the death of their partner due to persistent heterosexist and heteronormative attitudes in the UK [59]. Similar to research on cancer and LGBT+ populations, this small body of research suggests that culturally dominant norms surrounding cis-heteronormativity contributed to LGB people’s poor experience of palliative care and bereavement support. 

Palliative care research experts also emphasized the absence of large datasets as a barrier to developing evidence to improve palliative care and policy for LGBT+ populations. Furthermore, experts argued that palliative care more broadly was not on the national agenda of the current government. 

### 3.5. Mental Health

The review yielded 42 studies relating to LGBT+ mental health. Of these included papers, 10 were qualitative papers [65,66,67,68,69,70,71,72,73,74], 22 were quantitative papers [75,76,77,78,79,80,81,82,83,84,85,86,87,88,89,90,91,92,93,94,95,96], 6 were review papers [97,98,99,100,101,102], and 4 were mixed-methods papers [103,104,105,106]. Broadly, these papers were thematically divided across three areas. Firstly, six papers focused on professional opinions and treatment outcomes of LGBT+ mental health [70,77,82,87,98,99]. Secondly, 22 papers were concerned with the incidence of mental health problems, and the risk factors associated with the elevated rates of mental health problems within LGBT+ populations [75,76,78,79,80,81,83,84,85,86,90,91,92,93,94,95,96,97,100,101,102,106]. Thirdly, 14 papers were concerned with LGBT+ people’s experiences of having mental health problems and treatment, and explaining the factors contributing to their poor mental health [65,66,67,68,69,71,72,73,74,88,89,103,104,105].

A total of 22 of the studies examined young people’s experiences of mental health and consistently reported an increased incidence of LGBT+ youth poor mental health compared to their cis-heterosexual counterparts. Studies in mental health focused on conditions including depression, eating disorders, anxiety, suicidality and self-esteem, with no research conducted into exploring rarer diagnosis such as schizophrenia or dissociative identity disorder for example.

The evidence base is more developed in LGBT+ mental health inequality in the UK. This is facilitated by the recent inclusion of measures of sexual orientation (less so gender diversity) on large-scale datasets, e.g., the Adult Psychiatric Morbidity Survey [78]. This has produced some robust evidence such as a pooled analysis of 12 UK population surveys that demonstrates adults who identified as lesbian/gay have higher prevalence of common mental disorders when compared to heterosexual adults [91]. Importantly, the evidence base includes longitudinal datasets such as the Millennium Cohort Study that enable the tracking of poor mental health over the life course of LGBT+ populations. Amos and colleagues’ [75] study indicates that by age 10 years, depressive symptoms were higher in sexual minorities than in heterosexuals. Reflecting the greater availability of large-scale data, studies have been able to provide robust statistical analysis of the differences between identity groups particularly bisexual, trans, non-binary and gender non-conforming identities. In Colledge et al.’s study, for example, bisexual women were 37% more likely to have self-harmed compared to lesbians [79]. Rimes et al [88] found that female SAAB (sex assigned at birth) participants (aged 16–25) (binary and non-binary) were more likely to report a current mental health condition and history of self-harm than male SAAB participants (binary and non-binary).

The majority of papers utilised (implicitly) the minority stress theory [107] as a theoretical paradigm to explore the relationship between LGBT+ status and the increased risk of poor mental health. The minority stress theory is a psychological conceptual framework that is critiqued by some studies [108] as inadequate to explain the broader social and cultural norms that impact on LGBT+ people’s mental health. 

## 4. Discussion

This review of UK evidence aimed to describe what is known about LGBT+ health inequalities in relation to cancer, mental health, and palliative care to inform research, policy and public health interventions. The UK has a long tradition of research on health inequalities but until recently LGBT+ population groups were not included in public health research, policies and practice that attempt to tackle persistent health inequalities between population groups. Since the Equality Act 2010 [109], there has been a substantial increase in recognition that LGBT+ populations have a disproportionate health burden in comparison to cis-gendered heterosexual populations. This is evident in numerous health policies, e.g., Suicide Prevention Strategy [110], Transforming Children and Young People’s Mental Health Provision: a Green Paper [111]. In addition, the Equality Act 2010 public sector duty has driven a concern within the health care sector to demonstrate that it is providing services and care equally regardless of sexual orientation (and, to lesser degree, gender identity). There is much greater appetite from the UK Government, policy makers, NHS England, health staff and Public Health England to address LGBT+ health inequality. 

However, the findings from this scoping review suggest that the evidence base, on mental health, cancer and palliative care, is insufficient to address this nationally recognised health inequality. The current body of UK LGBT+ health inequality research is relatively small but there is clear evidence of health inequities between LGBT+ and cis-heterosexual populations. The research is strongest in terms of demonstrating the elevated rates of poor mental health in comparison to cis-heterosexual populations [91,110,112], reproductive and sexual health cancers, risk/transmission rates and barriers [113,114,115,116], and late diagnoses of cancer [2,117]. The evidence base for palliative care [4,7,8,63,64,118,119,120,121] is very small. Across all three areas of this review, there is consistent evidence that a significant proportion of health care providers are not well trained in LGBT+ identities, and consequently misunderstand the needs of LGBT+ populations [9,122]. This is particularly acute for trans and/or non-binary people who encounter difficulties engaging with gender identity services and health providers, and experience considerable barriers to reproductive and sexual health services [5,108,114,123].

The majority of LGBT+ health research in the three areas homogenise LGBT+ identities by using a single identity category, ‘LGBT’. This has the effect of obscuring health differences between identity categories. The notable exception is a small subset of LGBT+ mental health research where substantial population-based datasets are available for reliable statistical analysis of differences between identity categories. These studies have suggested that when compared to heterosexuals and people who identify as gay and/or lesbian, bisexual-identifying people experience more significant rates of suicidality, eating problems, self-harm, and addiction [79,112], and trans and/or non-binary people have significantly higher incidences of poor mental health and greater risk of suicide and self-harm [124]. Furthermore, little research examines the ways in which intersectional minoritised identities may mediate LGBT+ health inequality. There is a lack of exploration of race, ethnicity, faith, immigration status, social class, (dis)ability, aging, etc. This is a substantial omission in the evidence base to support public health interventions. The UK has decades of research that establishes the ways in which major social inequalities such as socio-economic status, gender and race/ethnicity are determinants of poor health. If LGBT+ health inequality is to be successfully addressed, then it is imperative that research consistently examines the intersectionality of LGBT+ health inequality. 

The results of this review suggest three key recommendations for the development of research on mental health, cancer and palliative care that can inform public health interventions to tackle LGBT+ health inequality. Firstly, there is often an absence of large datasets, with representative samples and administrative datasets on which to base our understanding of the extent of health inequality. Examples include differential cancer prevalence rates, access to health services, and treatment outcomes. Without large-scale datasets it is difficult to generate research that convinces policy makers and health care providers of the scale of the inequality. LGBT+ mental health research ‘weight’ of evidence has gained impetus at a national government level as large datasets have started to include measures of sexual orientation and gender identity, e.g., the Millennium Cohort Study, the Avon Bristol Study, the British Cohort Study 2012, the Health Survey for England 2011, 2012 and 2013, the Longitudinal Study of Young People in England 2009/10 and Understanding Society 2011/12. These are still missing for cancer and palliative care. 

Secondly, LGBT+ health inequality research has a tendency to ‘fix’ or essentialise identity. The impact of this is the homogenisation of the categories LGBT+, especially bisexuality and trans/non-binary. In most cases, research prioritises LGBT+ identity and ignores other interlocking systems of oppression/power relating to class, age, disability and race/ethnicity. Public health research and policy must work with a framework that has central the multiple experiences of inequality (race, gender class, etc.) that are mutually constitutive of health outcomes and experience for LGBT+ populations. It is crucial that if we want to address LGBT+ health inequality that we have reliable data on the ways in which health differs between LGBT+ people. 

Thirdly, the majority of LGBT+ health inequality research is conducted within the dominant frame of clinical, biomedical and lifestyle risk factors. Far less research employs an alternative theory using social models of health inequality such as socio-political, psychosocial or socio-ecological. The biomedical and lifestyle theories are individualistic approaches that primarily focus on decontextualised individual-level pathology, biology, and behaviour. In contrast, more social theories posit that there are interconnecting and complex social, cultural, political, economic factors that shape health inequality [15]. Much of the research included within our scoping review acknowledged that heteronormativity or LGBT+ discrimination and stigma were partly to blame for limiting access to services or poor experience of services. Very little UK research attempted to explain the underlying social mechanisms that influence LGBT+ health inequality. McDermott et al.’s [124] paper examining the social determinants of LGBT+ youth mental health inequality is a rare example (see also [120]). 

The dominance of the biomedical and lifestyle risk approach decontextualises LGBT+ health inequality and reduces the ways of addressing the problem to individual biological pathology and improving access to treatment and services. While clearly it is important to provide equity of access to health services and treatments, this does not, from a public health perspective, address the inequality between LGBT+ and cis-heterosexual populations. Why, for example, is there poorer mental health? Why are there low rates of cervical screening? The theories we use to understand LGBT+ health inequality impact on the questions we ask, the data we collect, our analysis, and eventually our ability to address LGBT+ health inequality at a population level [13]. The lack of alternative social theory leads to de-politicised descriptions of LGBT+ health inequality that do not recognise power and the unjust social relations that produce LGBT+ health inequities. This is a barrier to developing public health interventions that can successfully tackle LGBT+ health inequality.

The purpose of this review was to provide a tentative direction for LGBT+ health inequality research that would improve the ability of public health interventions to successfully reduce this inequality. A scoping review is a methodology that is used when there are few resources, to give an overview, and should not replace a more in-depth systematic review. Despite these methodological limitations, scoping reviews such as ours can be adaptable tools to direct future research that informs policy and practice. In our view, future research on LGBT+ health inequalities in mental health, cancer and palliative care needs to: (1) be resourced through large-scale datasets; (2) utilise theories/models that recognise the unequal social relations that produce health equality; (3) and pay attention to the health differences between LGBT+ populations.

## Figures and Tables

**Table 1:** Keywords used in Web of Science Searches.

Sexuality and Gender Keywords	Health Keywords	Location Keywords
Bisexual *	Mental disorder	United Kingdom
Gay	Mental * ill	UK
Lesbian	Mental * distress	Great Britain
Transgend *	Wellbeing	England
Same-sex	Psycholog *	NHS
Same-gender	Suicid *	
Sexual minorit *	Cancer	
Non-binary	Tumour	
Queer	Tumor	
Transsexual	Radiotherapy	
Asexual *	Chemotherapy	
Demisexual	Palliative	
Homosexual *	End of Life	
Pansexual *	Ag * care	
Two Spirit	Social care	
	Death	
	Dying	

**Table 2:** PubMed search strategy.

Sexuality and Gender ‘mesh’ Terms	Health ‘mesh’ Terms (Unless Specified as Keyword)	Location Keywords
Sexual and Gender minorities	Hospice and palliative care nursing	United Kingdom
Bisexuality	Palliative care	UK
Homosexuality	Palliative medicine	Great Britain
Transgender persons	Terminal care	England
	Death	NHS
	Bereavement	
	Grief	
	Home care services, hospital-based	
	Neoplasms	
	Cancer care facilities	
	Radiotherapy	
	Mental disorders	
	Psychology	
	Mental health	
	Suicid * (keyword in title)	

**Table 3:** Inclusion and exclusion criteria.

	Inclusion Criteria	Exclusion Criteria
Population	LGBT+ People	Non-LGBT+ People
Intervention	Those receiving treatment from the NHS or other publicly funded providers for any of the following: Cancer Palliative care Mental Health Those who are receiving social support due to end of life, mental health, or cancer. Public health approaches to: Cancer Palliative care Mental Health	Any other medical condition than those listed Papers focusing on secondary conditions arising from the primary point of interest
Comparison—not relevant for this study		
Outcome	All qualitative and quantitative data related to LGBT+ experiences and outcomes in cancer/mental health/palliative health and social care	Studies and findings related to other areas of health care Theoretical and conceptual pieces relating to this area
Study Details	Based on findings related to publicly funded health/social care providers All study designs (qualitative, quantitative, randomized control trials (RCTs), mixed method) Relevant grey literature and policy documents Published after 2005 English or Welsh language reporting	Published in languages other than English or Welsh Published before 2005 Opinion papers, dissertations, theses, newspaper articles, or editorials

## Appendix A

**Table A1:** Mental Health UK LGBT+ Studies.

Author, Year	Ref Number	Study Design/Aims/Objectives	Setting/Population/Sample	Methods	Main Results
Amos et al., 2016	[75]	To develop an up-to-date population-level estimate of the extent of risk across mental health problems, adverse social environments, and negative health outcomes between sexual minority adolescents and their heterosexual counterparts	9885 adolescents	Millennium cohort study analysis	Sexual minority adolescents in the UK experience disparities in mental health, social, and health-related outcomes despite living in a time of substantial progress in rights for sexual minorities. These adverse outcomes co-occur, with implications for lifelong health and social outcomes. Health and educational practitioners should be aware of the increased risk for adverse outcomes in sexual minority adolescents
Argyriou et al., 2020	[76]	To investigate psychosocial mediators in the association amongst young people between having a Lesbian, Gay, or Bisexual (LGB) orientation and depressive symptoms	14,814 individuals	Quantitative research; mediation analyses	Sexual minority youth (SMY) were at a higher risk for showing depressive symptoms at age 18 when compared to heterosexual young people SMY also had more unhelpful assumptions and poorer relationships with their family Suggest that more unhelpful self-beliefs, lower self-esteem, and poorer family relationships, contribute to higher amounts of depressive symptoms for SMY
Bartlett et al., 2009	[77]	To discover how professionals in psychotherapy and psychiatric organisations feel about treatments intended to change sexual orientation	1328 practitioners	Quantitative postal survey method	A significant minority of practitioners in psychology and psychiatric organisations are providing therapy to lesbian, gay, and bisexual clients to try and help them be heterosexual. These therapies do not have evidence that suggests they are effective
Bridge et al., 2019	[97]	To complete a systematic review and meta-analysis to highlight how sexual minority people’s self-esteem compares to heterosexual people	LGB people compared to heterosexual people	Systematic review and meta-analysis	Self-esteem is lower in sexual minority identified people when compared to heterosexual people Some evidence suggests that there may be even more differences for men and bisexual people, but this is not yet conclusive
Chakraborty et al., 2011	[78]	To see how mental disorder, self-harm, and suicide attempts compare between sexual orientations	7403 participants of the Adult Psychiatric Morbidity Survey	Quantitative statistical analysis based on a survey	Non-heterosexuals have a greater incidence of mental health problems and service usage Suggest that discrimination can act as a social stressor in the genesis of mental health problems
Colledge et al., 2015	[79]	To compare mental health between lesbian and bisexual women	937 bisexual women and 4769 lesbian women	Survey led quantitative methods	Bisexual women were more likely to report poor mental health or psychological distress when compared to lesbian women Suggest bisexual women have ‘double discrimination’ of homophobia and biphobia which is felt as internalised and stigma
Dhejne et al., 2016	[98]	To review literature relating to psychiatric disorders and psychopathology of trans people To review literature and the psychiatric outcome after gender-confirming interventions	Trans and gender non-conforming people	Review and synthesis	Trans people attending trans health care services seem to have more psychiatric morbidity which is improved following gender confirmation treatment
Foy et al., 2019	[103]	To explore LGB+ adult’s experiences with Improving Access to Psychological Therapies services and/or primary care counselling	136 LGBQ+ people	Mixed methods: online questionnaire and combination of descriptive statistics and thematic analysis	42% of LGBQ+ participants worried about experiencing LGBQ+-specific discrimination prior to accessing services 33% of clients did not disclose their sexuality, and 42% did not discuss sexuality in treatment LGBQ+ people felt that service stigma, lack of sexuality disclosure, lack of sexuality discussion in treatment, lack of LGBQ+ awareness affected their treatment 52% felt that treatment could be improved
Gabb et al., 2019	[65]	To see how LGBTQ+ young people could sustain or survive familial relationships	12 LGBTQ+ young people and 5 ‘family-like’ participants	Semi-structured interviews, emotion maps, and diaries	Families have a significant impact on LGBTQ+ young people’s lives and emotional landscapes Family practices can be paradoxical which need to be navigated by young people
Gnan et al., 2019	[80]	To observe LGBTQ-specific and general factors associated with having current mental health problems, and to look at the use of mental health services, and suicide risk and self-harm	1948 LGBTQ university students (age 16–25)	Survey analysis	All four outcomes were associated particularly with female gender, sexual abuse, other abuse or violence, and being trans Other more minimally associated factors (i.e., associated with one or more of the outcomes) included being bisexual, thinking that they were LGBTQ under the age of 10, coming out before age 16, not feeling accepted where they live, having no LGBTQ+ role models in universities, and experiencing LGBTQ-related crime
Hickson et al., 2016	[81]	To describe inequality in mental health indicators among gay and bisexual men	5799 men who are attracted to other men	Internet-based survey	Mental ill health common, particularly associated with lower education, younger age, and lower income Depression also associated with being a member of visible ethnic minority Depression also associated with being bisexual Cohabiting with a man and living in London were protective of mental health
Hughes et al., 2018	[82]	To examine how mental health staff perceive and practice working with LGBTQ youth suicide and self-harm in mental health care settings	113 NHS mental health staff across 3 trusts	Cross-sectional survey	Mental health staff have a decent knowledge about LGBTQ+ young people and self-harm and suicide Training is helpful to extend an understanding of sexual orientation Only a third of staff reported routinely discussing LGBTQ+ issues
Irish et al., 2018	[83]	To see the trajectory of depressive symptoms in sexual minority young people and heterosexuals from 10 to 21 To examine self-harm rates amongst heterosexual and sexual minority youths at ages 16 and 21	4828 adolescents	Longitudinal survey data	From the age of 10, depressive symptoms were more apparent in LGBTQ+ young people, and continued to increase with age to a larger extent LGBTQ+ people were more likely to report self-harm at ages 16 and 21 with no clear demonstration that this decreased with age
Jones et al., 2017	[84]	To investigate the risk of anxiety disorders in sexual minority youth and heterosexual youth To explore the influence of gender nonconformity, bullying, and self-esteem on anxiety disorders	5652 adolescents	Survey data	LGBTQ+ had higher earlier gender nonconformity, lower self-esteem, and reported more bullying than heterosexual adolescents Sexual minority youth are at increased risk of anxiety disorders relative to heterosexual youth at 17.5 years. Bullying between 12 and 16 years and lower self-esteem may contribute to this risk
King et al., 2007	[99]	To review existing research on counselling and psychotherapy for LGBTQ people To evaluate the contributions of different research techniques and identify future priorities	LGBT people in counselling and psychotherapy	Systematic and thematic review	Affirmative talking therapies help LGBT people counteract homophobia in early development The article concludes with many recommendations, some of which include: Psychotherapy training institutes need to include LGBT specific training, Encourage the training of LGBT therapists, Therapists should consider telling clients their own sexual identity
King et al., 2008	[100]	To identify the prevalence of mental disorder, substance misuse, suicide, suicidal ideation, and deliberate self-harm in LGB people	LGB people receiving counselling	Systematic review and meta-analysis	LGB people at higher risk of mental disorder, suicidal ideation, substance misuse, and deliberate self-harm than heterosexual people There are some differences between identities identified in this paper (e.g., LB women particularly at risk of substance use disorders)
Lewis, 2009	[101]	To identify any indicators or trends in relation to place, sexual minorities, and mental health	LGBT people	Meta-analysis	Policy regimes, health programming, and the ways in which sexual minorities are constructed in places all contribute to mental health in different spaces
Marshall et al., 2016	[102]	To assess the literature surrounding mental health problems (depression, NSSI behaviour and suicidal thoughts) and trans people	Trans people	Systematic review	There is a high prevalence of NSSI among trans people—particularly trans men—when compared to cis people Suicidality is higher amongst trans people than cis people
McAndrew and Warne, 2010	[66]	To gain an in-depth understanding of life experiences leading to suicidality amongst four gay men	4 gay men	Psychoanalytically informed interviews	Significant life experiences revealed why some men may have both long term mental health problems and also engage in suicidality (father/son relationship, loneliness of outsiderness, leading a double life are some examples)
McAndrew and Warne, 2012	[67]	To offer an understanding of gay men’s experiences of mental well-being	4 gay men	Psychoanalytically informed interviews	Heterosexism affects the development of gay children, making it harder for them There is a necessity for nurturing that extends beyond heterosexist assumptions in schools
McDermott, 2006	[68]	To examine the ways in which the psychological health of women may be influenced by workplace sexual identity performances and social class positioning	24 lesbian or gay women	Semi-structured interviews	Workplace settings are a significant factor in lesbian/gay women’s psychological health Social class can mediate the risk of compulsory heterosexuality in the workplace, where middle class women are best positioned to negotiate their sexuality in the workplace
McDermott, 2015	[69]	To see how LGBT young people seek help in relation to their mental health	LGBT people in online spaces	Qualitative virtual methods	Young LGBT often want assistance to support their mental health, but find it difficult to ask for help, articulate their distress and tell people that they are ‘failed’ There is a lot of shame associated with negotiating norms of heterosexuality and adolescence These norms regulate what emotions it is possible to articulate and what lives are therefore possible
McDermott et al., 2018	[104]	To explore how LGBT young people seek help and experience suicidality	29 LGBT young people (interviews) and 789 young people (surveys)	Interviews and an online survey	Participants only ask for help when they reach a crisis point as they normalise their distress Seeking help is more likely when connected to self-harm, suicidality or sexuality/gender based abuse The reluctance to seek help comes from negotiating norms and being unable to talk about emotions
McDermott et al., 2018	[105]	To investigate the social determinants of the mental health inequality amongst LGBT young people and heterosexuals	29 LGBT young people (interviews) and 789 young people (surveys)	Interviews and an online survey	Social determinants included homo/bi/transphobia, sexual and gender norms, managing sexual and gender identity across different life domains, being unable to talk, other life crises Some identities were more likely to be suicidal, including trans youth, disabled youth, those affected by abuse, and those with a previous history of self-harm
Oginni et al., 2019	[85]	To investigate self-esteem and depressive symptoms as mediators of increased rates of suicidal ideation or self-harm among sexual minority youth, and the roles of childhood gender nonconformity and sex as moderators of these relationships	4724 young people	Survey data	SMY were 3x more likely to report past year suicidal ideation or self-harm Lower self-esteem and increased depressive symptoms partly explain the increased risk for later suicidal ideation and self-harm in SMY
Page et al., 2016	[70]	To report on one health board’s attempts to work more closely with older members of the transgender community	Older trans people with mental health problems accessing specialist services	Appreciative inquiry to deliver service chance	Nurses learned a lot more about trans issues through the course of this research Older trans people met receptive workers in the organisation Further change might happen
Rasmussen et al., 2019	[86]	To use the Integrated Motivational Volitional Model of Suicide (IMV) to examine sexual orientation To examine demographics, psychological distress and personality as they impact the IMV motivational factors of defeat, entrapment and suicidal ideation	418 18–34 year olds	Cross-sectional online survey	Results showed that there were high rates (>50%) of a 12 month suicidal ideation prevalence and also (>45%) a willingness to enact a future suicide attempt Bisexual (and pansexual) reported high levels of IMV-related outcomes (for example internal entrapment or defeat)
Rimes et al., 2018	[87]	To compare socio-demographic and clinical characteristics with treatment outcomes across sexual orientation groups amongst adults receiving primary psychological care interventions	188 lesbians, 222 bisexual women, 6637 heterosexual women, 645 gay men, 75 bisexual men, and 3024 heterosexual men	Clinical data used to compare treatment characteristics	Varying differences between identity groups Lesbian and bisexual women (particularly bisexual women) show less benefit from primary psychological intervention compared with heterosexual women No significant difference for men, but was not enough data to conclude this entirely for bisexual men
Rimes et al., 2019	[88]	To compare mental health, self-harm, and suicidality, and substance use, and victimisation experiences between non-binary and binary trans young adults	105 trans females, 210 trans males, 93 non-binary AMAB, 269 AFAB non-binary	Online survey data	AFAB were more likely to report self-harm and a current mental health condition than AMAB AFAB participants more likely to report sexual abuse Non-binary AMAB were less likely to report past suicide attempts and previous help seeking for depression/anxiety Binary participants reported lower life satisfaction than non-binary participants Mental health problems, self-harm, suicidality, alcohol use, and victimisation experiences all higher when compared to general population statistics
Rimes et al., 2019	[90]	To investigate LGB related and other factors associated with suicide attempts, suicidal ideation, and future suicide risk	3575 LGB young adults	Survey	LGB stigma and discrimination were significantly associated with suicide attempts, ideation, and future suicide attempt likeliness Bisexuality, not feeling accepted where one lives, younger sexual minority identification, and younger coming out were also associated with suicidality Other associated factors included female gender, lower social support, anxiety/depression help seeking, abuse/violence, and sexual abuse.
Rimes et al., 2019	[89]	To investigate whether sexual minority patients have poorer treatment outcomes than heterosexual patients	Patients attending Improving Access to Psychological Therapies (IAPT) services	Routine national data	Lesbian and bisexual women have higher final session severity for depression, anxiety, and functional impairment, and increased risk of not attaining reliable recovery Bisexual men had higher final session severity for depression, anxiety, and functioning and increased risk of not attaining reliable recovery when compared to heterosexual and gay men Gay and heterosexual men did not differ on treatment outcomes Racial minority LGB patients did not have significant outcomes compared to white LGB patients
Rivers et al., 2018	[71]	To explore participants’ narratives surrounding their perception of risk and protective circumstances To explain suicide attempts in youth	17 LGBT individuals	Interviews	Some LGBT individuals have effectively—although arduously—navigated suicidal crises using various coping methods
Roen et al., 2008	[72]	To understanding how young people make sense of suicide	69 participants aged 16–24	Interviews and focus groups	Young people understand suicide as: suicidal subjects as other, suicide as accessible, rationalising suicidal behaviour, defining suicidal subjects in terms of their relationships with others
Scourfield et al., 2011	[73]	To see how young people talk about self-harm	13 LGBT interviewees, 66 LGBT focus groups	Interviews and focus groups	Dichotomy between display for public and personal intense distress amongst LGBT people Self-harm can be private, but can be a way of reaching out Self-harm is complicated and multifaceted
Scourfield et al., 2008	[74]	To explore cultural context of youth suicide To explore connections between sexual identity and self-destructive behaviour	69 young people	Interviews and focus groups	LGBT young people employ resilience, ambivalence and self-destructive behaviour when faced with distress Practitioners should move to ecological approach to help LGBT young people
Semlyen, J er al., 2016	[91]	To determine an estimate of the association between sexual orientation identity and poor mental health and well-being among adults from 12 population surveys in the UK, and to consider whether effects differed for specific subgroups of the population	94,818 participants of large-scale surveys	Survey analysis	In the UK, LGB adults have higher prevalence of poor mental health and low well-being when compared to heterosexuals, particularly younger and older LGB adults. Sexual orientation identity should be measured routinely in all health studies and in administrative data in the UK in order to influence national and local policy development and service delivery. These results reiterate the need for local government, NHS providers and public health policy makers to consider how to address inequalities in mental health among these minority groups
Sherr et al., 2008	[106]	To measure suicidal ideation in HIV clinic attenders in the UK	778 attendees at five HIV clinics	Questionnaire, clinic note data, treatment data	31% prevalence of suicidal ideation Factors associated with suicidal ideation included being heterosexual man, black ethnicity, unemployment, lack of disclosure of HIV status, stopping antiretroviral treatment, psychical and/or psychological symptoms, and poorer quality of life
Taylor et al., 2018	[92]	To explore the association between LGB status and self-harm in UK higher education students	707 UK university students	Online survey	LGB status associated with an elevated risk of NSSI and SA
Timmins et al., 2017	[93]	To test direct/indirect associations between minority stressors and psychological distress among trans people	1207 trans individuals	Online survey	There is a relationship between minority stressors and psychological distress among trans people These relationships are partially explained by rumination
Timmins et al., 2018	[94]	To investigate minority stressors and distress in twins To understand the role of the environment	38 twin pairs, where one is heterosexual and the other is LGB	Questionnaire survey	LGB twins have high rumination LGB twins high incidence of rejection, active concealment, self-stigma, prejudice events, childhood gender non-conformity, and lower scores on sexual orientation disclosure Environmental factors are a causal explanation for disparities in rumination between LGB and heterosexual individuals
Timmins et al., 2019	[95]	To test mechanisms by which social stigma contributes to psychological distress in sexual minority individuals	4248 LGB people	Survey	Strong relationship between minority stressors and psychological distress in LGB individuals, which are partially accounted for by rumination
Woodhead et al., 2016	[96]	To compare well-being, common mental disorder symptoms, suicidal ideation over a lifetime, alcohol and drug use amongst non-heterosexual and heterosexual individuals	7403 adults	Survey	Non-heterosexual orientation strongly associated with common mental disorder, lifetime suicidal ideation, harmful alcohol and drug use Inner city sample had poorer mental health overall compared with national sample—this discrepancy was larger for non-heterosexuals than heterosexuals Childhood and adult adversity substantially influence but do not account for sexual orientation related mental health disparities

**Table A2:** UK Cancer and LGBT+ Studies.

Author, Year	Ref Number	Study Design/Aims/Objectives	Setting/Population/Sample	Methods	Main Results
Alexis and Worsley, 2018	[23]	To explore gay and bisexual men’s experience of prostate cancer post-treatment	Gay and bisexual men in prostate cancer post-treatment	Meta-synthesis	Gay and bisexual men experience sexual impact, physical, and psychological difficulties, as well as challenges to intimacy and support mechanisms. The systems in place need to proactively accommodate gay and bisexual men
Burkhaltler et al., 2016	[37]	To identify gaps in LGBT cancer research	2 day Summit on Cancer in LGBT Communities	56 summit participants	Shared lessons and experience resulting in 16 recommendations covering sexual orientation and gender identity data collection, clinical care of LGBT persons, and education and training of health care providers
Czoski-Murray et al., 2010	[24]	To assess cost effectiveness of screening for anal cancer in men and women who are HIV positive, and in particular, men who have sex with men	People with anal cancer	Cost-effectiveness screening	It is unlikely that screening of identified high-risk groups will generate health improvements at a reasonable cost
Dalla Pria et al., 2014	[25]	To assess the efficacy of screening for potential anal cancer based on presence of anal lesions in HIV-positive MSM	HIV-positive MSM undergoing anoscopy screening	High resolution anoscopy with intervention for high grade squamous intrapithetial lesions was offered to asymptomative HIV-positive MSM. Patients and HSILs were treated and follow-up HRA was performed after 6 months whilst patients with low-grade squamous intrapethelial lesions had a repeat HRA after 12 months	AIN-3 is a significant risk factor for subsequent anal cancer, although the tumours detected in screened patients were small localised, and generally the outcomes were favourable
Darwin and Campbell, 2009	[35]	To assess sexual minority women’s opinions relating to cervical screening	34 sexual minority women	Q sorts	There is a need for affirmation of diversity within criteria for national screening programmes. There is a complexity of meanings around cervical screening
Doran et al., 2018	[26]	To understand gay men’s experiences of health care (all cancer-related)	Gay men with experience of prostate cancer	Interviews	Participants wanted, and expected, candid discussions with health care professionals about how prostate cancer could affect their lives, sexual function, and how to access culturally relevant support before and after treatment. Participants perceived that their health care team had little knowledge about their needs, and if, or how, their experience differed due to their sexual orientation. Information provided was considered heteronormative
Fish, 2016	[42]	To conduct a PAR project with the hope of improving LB women’s experiences of breast cancer health care	Lesbian and bisexual women	PAR-informed knowledge and exchange programme	This project raised awareness of unmet need in relation to LB women with breast cancer; this in itself was notable as their needs were previously assumed to be the same as those of their heterosexual counterparts. Subsequently, LB women were included in equality and diversity policy statements for the first time. The project also provided a number of different learning opportunities for cancer professionals to develop knowledge, attitudes and skills
Fish and Anthony, 2005	[44]	To see whether risk perceptions, experiences of health care, and health seeking behaviour were correlated	1066 lesbians	Survey	While lesbians were less likely than lesbians in a similar U.S. study to report that their risk of cervical cancer was the same as that of heterosexual women, perceptions of risk were not correlated with participation in screening. We assumed that bad experiences of screening would act as a barrier to attendance; instead, good experiences were associated with the increased likelihood of attendance. These findings underscore the need for a pro-active agenda for lesbian health which addresses the need for culturally competent health care, the sharing of best practice amongst health care providers, and the creation of systemic institutional change to improve the care lesbians receive
Fish and Williamson, 2018	[38]	To find out experiences of LGB people with cancer	15 British LGB cancer patients	Interviews	There is the ‘awkward choreography around disclosure’ opportunities and dilemmas for LGB patients, we describe ‘making sense of sub-optimal care’ which included instances of overt discrimination but was more frequently manifested through micro-aggressions and heteronormative systems and practices, and explore accounts of ‘alienation from usual psychosocial cancer support’
Fish et al., 2019	[39]	To explore conditions under which a sample of British LGB cancer patients revealed their sexual orientation in hospital settings to enable a more nuanced approach to understanding disclosure in this context	30 LGB patients with cancer	Semi-structured interviews	There are three themes as part of the analysis: authenticity as a driver for disclosure in cancer care; partners as a (potential) salutogenic resource; and creating safe, healing environments conducive to disclosure. The findings are reported and discussed in relation to three inter-related concepts from current salutogenesis theorising including a sense of coherence, generalised resistance resources and healing environments which can facilitate sexual orientation disclosure
Fox et al., 2009	[55]	To determine whether imiquimod was more effective than placebo for the treatment of high-grade anal canal intraepithelial neoplasia (HG-ACIN)	64 HIV-positive MSM	Double-blind, randomised placebo-controlled clinical trial. Sixty-four HIV-positive patients were randomised to self-application of imiquimod cream or matched placebo into the anal canal three times a week for 4 months. Response was assessed by cytology, high-resolution anoscopy and biopsy 2 months after therapy. All patients who failed to resolve were offered treatment with open-label imiquimod for a further 4 months	This study demonstrates the effectiveness of imiquimod for the treatment of ACIN, and the benefit of prolonged or repeated treatments. This form of therapy is likely to be especially valuable for patients with widespread multifocal ACIN who are otherwise difficult to treat, and should be considered as an adjunct to ablative therapy
Henderson, 2009	[36]	To explore the evidence base for the premise that women who are exclusively lesbian have a very low chance of developing cervical cancer	Lesbians	Literature review	Case reports and prevalence studies show that HPV can be transmitted sexually between women. It is not known whether prevalence of HPV or cervical cancer differs between lesbians and heterosexual women. The evidence consistently shows that prevalence of non-attendance for cervical screening is much higher in lesbian than heterosexual women, which is linked to a belief that lesbians are less susceptible to cervical cancer and have less need for screening. Despite sharing most of the same risk factors as heterosexual women, lesbians are much less likely to undergo regular screening
Heyworth, 2016	[28]	To conduct a prostate cancer awareness survey, the results of which would inform the development of new patient information	217 gay/bisexual men and trans women	Survey	Using the data from the survey and the available evidence base, it was agreed that four postcards should be produced, addressing four key areas: survivorship—and specifically issues around sexual function following treatment; the prevalence of prostate cancer in gay and bisexual men; taking care of your prostate—reducing cancer risk; communication
Hulbert-Williams et al., 2017	[40]	To explore cancer experiences in LGB people	68,737 individuals (0.8% were lesbian, gay, or bisexual)	National Cancer Patient Experience Survey	There is a pattern of inequality, with less positive cancer experiences reported by lesbian, gay and (especially) bisexual respondents. Poor patient–professional communication and heteronormativity in the health care setting potentially explain many of the differences found. Social isolation is problematic for this group and warrants further exploration
Karnon et al., 2008	[29]	Cost–utility analysis of screening for anal cancer in high-risk groups from a UK perspective	MSM	Cost utility analysis	Reference case results showed screening is unlikely to be cost effective. Sensitivity analyses identified two important parameters: regression from low-grade anal intraepithelial neoplasia (AIN) and utility effects. Increased AIN regression rates resulted in a minimum incremental cost per QALY gained of £39 405, whereas a best case scenario reduced the ratio to £20 996.
Kelly et al., 2018	[30]	To discuss the risks that heteronormative assumptions play in prostate cancer care and how these may be addressed	LGBT patients with cancer	Identification and inclusion of relevant international evidence combined with clinical discussion	This paper posits a number of questions around heteronormativity in relation to prostate cancer information provision, supportive care and male sexuality. While assumptions regarding sexual orientation should be avoided in clinical encounters, this may be difficult when heteronormative assumptions dominate. Existing research supports the assertion that patient experience, including the needs of LGBT patients, should be central to service developments.
Kesten et al., 2019	[45]	To understand young MSM’s knowledge and attitude towards HPV vaccination	51 YMSM completed questionnaires and 17 YMSM participated in focus groups	Questionnaires and focus groups	Over half of YMSM were aware of HPV (54.9%), yet few (21.6%) had previously discussed vaccination with a health care professional (HCP). Thematic analyses found that YMSM were willing to receive the HPV vaccine. Vaccination programmes requiring YMSM to request the vaccine, particularly prior to sexual orientation disclosure to family and friends, were viewed as unfeasible. Educational campaigns explaining vaccine benefits were indicated as a way to encourage uptake
King et al., 2015	[46]	To estimate the prevalence of oral detectable HPV DNA in HIV-negative MSM attending a sexual health clinic in London and concordance with anogenital HPV infection	151 HIV negative MSM	Paired oral rinse samples and anogenital samples were available from 151 HIV-negative MSM within a larger cross-sectional survey. All samples were tested in parallel for 21 types of HPV DNA using an in-house assay	HR-HPV DNA, including HPV 16/18, was detected in oral specimens from HIV-negative MSM attending sexual health clinics, suggesting a potential role for vaccination, but is far less common than anogenital infection. How this relates to the risk and natural history of HPV-related head and neck cancers warrants further study. Lack of concordance with anogenital infection also suggests that oral HPV infection should be considered separately when estimating potential vaccine impact
King et al., 2015	[47]	To see whether HPV prevalence and risk factors in MSM could inform the potential effectiveness of vaccinating MSM from HPV	522 MSM aged 18-40	Cross-sectional study of 522 MSM aged 18–40 attending a London sexual health clinic who completed a computer-assisted self-interview. Urine and two swabs (anal and penile/scrotal/perianal) were collected and tested using an in-house Luminex-based HPV genotyping system.	On the basis of the current infection status, most MSM, even among a high-risk population attending a sexual health clinic, are not currently infected with the vaccine-type HPV. A targeted vaccination strategy for MSM in the UK could have substantial benefits
Matheson et al., 2017	[31]	To synthesise existing qualitative research on younger/unpartnered/gay men to see how prostate cancer impacts these groups	Young, unpartnered, gay/bisexual men with prostate cancer	Systematic meta-synthesis	The three overarching constructs illustrated the magnified disruption to men’s biographies, namely marginalisation, isolation and stigma—relating to men’s sense of being “out of sync”; the burden of emotional and embodied vulnerabilities and the assault on identity, illustrating the multiple threats to men’s work, sexual and social identities; shifting into different communities of practice—such as the shift from being part of a sexually active community to celibacy
McConkey and Holborn, 2018	[32]	To explore the lived experience of gay men with prostate cancer	8 gay men treated for prostate cancer	In-depth interviews	Gay men with prostate cancer have unmet information and supportive care needs throughout their prostate cancer journey, especially related to the impact of sexual dysfunction and associated rehabilitation, negatively impacting their quality of life. Issues associated with heteronormativity, minority stress, and stigma may influence how gay men interact with the health service, or how they perceive the delivery of care. Health care education providers should update prostate cancer education programmes accordingly
Meads and Moore, 2013	[43]	This systematic review investigated all evidence on whether there is, or likely to be, higher rates of breast cancer in LB women	Lesbians and bisexual women	Systematic review	Searches found 198 references. No incidence rates were found. Nine studies gave prevalence estimates - two showed higher, four showed no differences, one showed mixed results depending on definitions, one had no comparison group and one gave no sample size. All studies were small with poor methodological and/or reporting quality. One incidence modelling study suggested a higher rate. Four risk modelling studies were found, one Rosner-Colditz and three Gail models. Three suggested higher and one lower rate in LB compared to heterosexual women. Six risk factor estimates suggested higher risk and one no difference between LB and heterosexual women
Merriel et al., 2018	[48]	To explore and compare the knowledge and attitudes of UK General Practitioners and sexual health care professionals regarding HPV vaccination for YMSM (16–24)	GPs and Sexual Health Care Practitioners	Survey	Twenty-two participants (20 SHCPs, *p* < 0.001) had vaccinated a YMSM patient against HPV. GPs lack of time (25/38, 65.79%) and SHCP staff availability (27/49, 55.10%) were the main reported factors preventing YMSM HPV vaccination. GPs were less likely than SHCPs to believe there was sufficient evidence for vaccinating YMSM (OR = 0.02, 95% CI = 0.01, 0.47); less likely to have skills to identify YMSM who may benefit from vaccination (OR = 0.03, 95% CI = 0.01, 0.15); and less confident recommending YMSM vaccination (OR = 0.01, 95% CI = 0.00, 0.01). GPs appear to have different knowledge, attitudes, and skills regarding YMSM HPV vaccination when compared to SHCPs
Nadarzynski et al., 2017	[49]	To identify sexual health care professionals perceived barriers and facilitators MSM-targeted MSM HPV vaccination	13 doctors, 3 nurses, 3 health care advisors	Telephone interviews	HCPs were unsure about selection criteria, acceptable health care settings and the source of vaccination funding for the introduction of MSM-targeted HPV vaccination. Lack of political and public support, MSMs’ limited access to HPV vaccination and disclosure of sexual orientation to HCPs, identification of eligible MSM, patients’ poor HPV awareness and motivation to complete HPV vaccination were perceived as significant barriers. HCPs believed that the introduction of official guidelines on HPV vaccination for MSM, awareness campaigns and integrated clinic procedures could improve vaccination coverage
Nadarzynski et al., 2018	[50]	To examine HPV vaccine acceptability amongst MSM in the UK	1508 MSM	Online survey	Although nearly half of MSM would not actively pursue HPV vaccination, the vast majority would accept the vaccine if recommended by HCPs. In order to achieve optimal uptake, vaccine promotion campaigns should focus on MSM who do not access SHCs and those unwilling to disclose their sexual orientation
Nadarzynski et al., 2017	[51]	To explore MSMs perceptions of HPV and HPV vaccination prior to the introduction of this programme	33 MSM	Focus groups and interviews	Most MSM have poor knowledge about HPV and associated anal cancer. Despite the lack of concern about HPV, most MSM expressed willingness to receive HPV vaccination. There is a need for health education about the risks of HPV and HPV-related diseases so that MSM can appraise the benefits of being vaccinated. Concerns about HPV vaccine effectiveness in sexually active men and possible stigmatisation need to be addressed to optimise HPV vaccine acceptability
Nadarzynski et al., 2015	[52]	To explore MSMs perceptions of HPV and HPV vaccination prior to the introduction of this programme	325 sexual health professionals	Survey	Among 325 sexual health professionals, 14% were already vaccinating men against HPV, 83% recommended gender-neutral HPV vaccination and 65% recommended targeting MSM. Over 50% reported having poor knowledge about the use of HPV vaccine for MSM and the skills to identify MSM likely to benefit from HPV vaccination
Ong et al., 2014	[54]	To assess the literature to see how regular digital anorectal examination (DARE) is incorporated into HIV management guidelines	HIV-positive MSM	Systematic review	Few HIV guidelines discuss or recommend DARE as a means of anal cancer screening. Studies of the efficacy, acceptability and cost effectiveness of DARE are needed to assess its role in anal cancer screening
Saunders et al., 2017	[41]	To address gaps in evidence on the risk of cancer in people from sexual minorities	769,594 people from English GP Patient Survey and 249,010 people from English Cancer Patient Experience Survey	Survey	Large-scale evidence indicates that the distribution of cancer sites does not vary substantially by sexual orientation, with the exception of some HPV- and HIV-associated cancers. These findings highlight the importance of HPV vaccination in heterosexual and sexual minority populations
Schofield et al., 2016	[33]	To establish the feasibility and acceptability of anal screening among MSM	Known HIV-positive and negative MSM who have anoreceptive intercourse	Anal screening with human papilloma virus (HPV) testing, liquid-based cytology and high-resolution anoscopy with biopsy of anoscopic abnormalities. Participants completed questionnaires at baseline and at 6 months	The high prevalence of high-risk HPV and frequency of false negative cytology in this study suggest that high-resolution anoscopy would have most clinical utility, as a primary screening tool for anal cancer in a high-risk group. The prevalence of AIN3+ in HIV-positive MSM lends support for a policy of screening this group, but the high prevalence of lower grade lesions which do not warrant immediate treatment and the limitations of treating high-grade lesions requires careful consideration in terms of a screening policy.
Soe et al., 2018	[53]	To investigate the cost effectiveness for HPV vaccination program in older women (age > 26), heterosexual men, and MSM	Older women, heterosexual men, and MSM	Literature review	Targeted HPV vaccination for MSM should be next priority in HPV prevention after having established a solid girls vaccination programme. Vaccination for heterosexual men should be considered when 2-dose 4vHPV/9vHPV vaccines become available with a reduced price, whereas targeted vaccination for women over age 26 is unlikely to be cost effective

**Table A3:** Palliative UK LGBT+ Studies.

Author, Year	Ref Number	Study Design/Aims/Objectives	Setting/Population/Sample	Methods	Main Results
Almack et al., 2010	[57]	To see how sexual orientation may impact on concerns about and experiences of end of life care and bereavement within same-sex relationships	Lesbian and gay elders	Focus groups	The focus on end of life care and bereavement sheds light on a series of relatively neglected issues associated with lesbian, gay and bisexual (LGB) ageing and, more broadly, the topics of care and support within ‘non-traditional’ intimate relationships and personal networks. They point to the importance of further research into the lives of older lesbians and gay men facing issues of end of life care and bereavement
Almack et al., 2015	[63]	To identify particular needs salient to sexual/gender orientation relating to end of life care as identified by older LGBT people To examine how sexual and gender orientation may impact on experience of end of life care for LGBT older people To explore LGBT older people’s familial and friendship networks and the ways in which these personal networks may influence later life experiences towards and at end of life To identify recommendations for good practice in end of life care for older LGBT people	Older LGBT people	Survey, interviews, and public engagement workshop	LGBT people wanted greater inclusivity, a reflection of diversity, validation, safety, and ‘like-minded’ people in accessing services. Those working in end of life care should be sensitive to understanding LGBT lives. Trans people had specific concerns around being misgendered after their deaths
Almack, K 2018	[8]	To explore the end of life care experiences and care needs of older LGBT people aged 60+	LGBT+ people aged 60+ or LGBT people in a relationship with someone aged 60+ across the UK	Survey and interviews	Participants’ networks presented a complex diversity and richness including families of origin and of choice. It is clear however that LGBT older adult’s histories and pathways have ongoing profound influences on the means of social support available to individuals at the end of life
Almack et al., 2015	[62]	To explore inequalities experienced by LGBT people accessing palliative and end of life care services	Older LGBT people	Stakeholder meetings and focus groups	It is important to look at the whole person and address heterosexist attitudes in the delivery of end of life care. Developing cultural sensitivity in addressing the distinct, complex and multiple needs of LGBT people holds the potential to develop non-discriminatory services that will benefit everyone
Bristowe et al., 2016	[64]	To identify and appraise the evidence of the bereavement experiences of lesbian, gay, bisexual, and/or trans* people who have lost a partner and develop an explanatory model of lesbian, gay, bisexual and/or trans partner bereavement	LGBT people who have lost a partner	Systematic review	Studies described universal experiences of the pain of losing a partner; however, additional barriers and stressors were reported for lesbian, gay, bisexual and/or trans* people, including homophobia, failure to acknowledge the relationship, additional legal and financial issues and the ‘shadow’ of HIV or AIDS. A novel model was developed to explain how the experience for lesbian, gay, bisexual and/or trans* people is shaped by whether the relationship was disclosed and acknowledged in life and into bereavement and how this impacts upon needs and access to care
Bristowe et al., 2018	[61]	To explore health care experiences of LGBT people facing advanced illness	40 LGBT people from across the UK facing advanced illness (cancer = 21, non-cancer = 16, and both cancer and non-cancer = 3)	Semi-structured interviews	Experiences of discrimination and exclusion in health care persist for LGBT people. Ten recommendations are made within this data
Fenge, 2013	[58]	To consider the experience of loss and bereavement for lesbian and gay elders	Bereaved lesbian and gay elders (4) and an agency that works with older LGBT people	Interviews	Issues include: undisclosed identities, lack of recognition of partnership, disenfranchised grief, and cultural competency within health and social care service workforce, and accessing appropriate bereavement services, and support from funeral homes
Harding et al., 2012	[7]	To identify and appraise the existing evidence for the needs, experiences, and preferences for palliative and end-of-life care in lesbian, gay, bisexual and transgender (LGBT) populations	12 articles, primarily relating to cancer experience of gay and lesbian people	Systematic review	Existing evidence is explicit and indeed repetitive in highlighting the educational needs of health care professionals to explore sexual preferences, avoid heterosexist assumptions, and recognise the importance of partners in decision making. There is also a significant need to research LGBT experiences and refine services for patients and their caregivers
Ingham et al., 2016	[59]	To explore experiences of same-sex partner bereavement in women over the age of 60	8 women who had lost a same-sex partner	Semi-structured interviews and an interpretative phenomenological analysis	The findings indicate that in addition to the experiences of partner bereavement noted in research with heterosexual widows, older women who lose same-sex partners may face particular challenges, which can impact upon psychological well-being and adjustment to loss. These challenges appear to result from past and current homophobic and heterosexist attitudes within the UK culture. A range of interventions at individual, group, health service, and societal levels may be beneficial in improving the psychological well-being of older women who lose a same-sex partner
Westwood, 2017	[60]	To explore the ’right to die’ debate from the perspectives of older lesbians and gay men	60 older LGB individuals	Semi-structured interviews	Older lesbians and gay men are multiply disadvantaged (a) by an increased risk of feeling that life is not worth living due to affective inequalities (inadequate informal and formal social support) and (b) by a denial of access to the right to die both under such circumstances and/or if they wish to resist the normativities associated with a passive, medicalised death. There is a need to distinguish between a wish to die because of deficiencies in the care system and a wish to die in order to control how, when and where one’s life ends. The analysis highlights the contextual contingencies of ‘vulnerability’ in relation to the right to die and interrogates the heterosexist and disciplinary reproductive normativities underpinning the notions of ‘natural’ deaths
